# Association of the superior semicircular canal and tegmen tympani dehiscences and its relationship with the pneumatisation of the temporal bone

**DOI:** 10.1007/s00405-023-08243-y

**Published:** 2023-09-30

**Authors:** J. Whyte-Orozco, A. I. Cisneros-Gimeno, A. García-Barrios, M. E. Lozano-Langarita, A. Whyte-Orozco, E. Rubio-Aranda

**Affiliations:** 1 Department of Human Anatomy and Histology, School of Medicine, University of Zaragoza, C/ Domingo Miral, S/N, 50009 Zaragoza, Spain; 2 Medical and Genetic Research Group (GIIS099) Aragon Health Research Institute, Zaragoza, Spain; 3 Antecessor B51_23D (Government of Aragon), Zaragoza, Spain; 4 Department of Animal Pathology, School of Veterinary, University of Zaragoza, Zaragoza, Spain; 5 Department of Microbiology, Pediatrics, Radiology and Public Health, School of Medicine, University of Zaragoza, Zaragoza, Spain; 6 Water and Environmental Health /B43_23R, Zaragoza, Spain

**Keywords:** Superior semicircular canal dehiscence, Tegmen tympani dehiscence, Temporal bone, Pneumatisation

## Abstract

**Purpose:**

To analyse the degree of pneumatisation of the temporal bone when there is an association between dehiscence of the superior semicircular canal and dehiscence of the tegmen tympani.

**Materials and methods:**

We analysed a retrospective CT study of 124 selected cases. A single inclusion criterion was applied: the presence of a dehiscence of the tegmen tympani. On the other hand, the degree of temporal pneumatisation was assessed by axial and coronal planes, and has been divided into the following grades O, I, II and III, according to the status and relationship of the mastoid, the bony labyrinth, the petrous segment of the carotid canal and sigmoid sinus.

**Results:**

Of the 124 cases studied, 35 (28.2%) presented both dehiscences. In 26 of the 35 (47.3%), grade II pneumatisation, 4 (14,8%), grade I, and 5 (11,9%) grade III was observed, with a statistically significant relationship (*p* < 0.001). On the other hand, we did not find a significant relationship when relating both dehiscences in any age or sex group. However, when relating the degree of pneumatisation to sex, among those with grade III pneumatisation, the proportion of men (52.4%) was significantly higher than that of women (47.6%) (*p* = 0.017).

**Conclusion:**

We have detected a statistically significant relationship between the coexistence of grade II pneumatisation and the presence of both dehiscences in the temporal bone.

## Introduction

Minor first observed the relationship between tegmen tympani dehiscence and that of the superior semicircular canal in 2000 [1] in three cases of dehiscence of the superior semicircular canal that he treated surgically and that, in addition, presented damage to the tegmen.

The association between both dehiscences is strongest within the otic capsule syndrome, with an incidence ranging from 13.04% reported by Cloutier to 76% described by Nadaraja [2, 3].

Given the increased likelihood of both defects, it is recommended that when one is diagnosed, the possibility of the other should be investigated, especially if otological meningitis or conduction hearing loss is present, before exploratory tympanic surgery or linked to vestibular symptoms [4–6].

Although the literature has described the relationship between pneumatisation of the temporal bone in patients with superior canal dehiscence [7–10], we are unaware of any study that relates the presence of both bone defects to the degree of pneumatisation; thus, investigated the potential relationship between the coexistence of a certain type of pneumatisation and the presence of both dehiscences.

## Materials and methods

The registry of patients was obtained from the database of the research group Medical and Genetic Research (GIISO99) from five tertiary Spanish hospitals: Basurto and Cruces Hospitals (Bilbao, Spain), General Hospital of the Defense, Miguel Servet and Lozano Blesa Hospitals (Zaragoza, Spain). A single inclusion criterion was applied: the presence of a dehiscence of the tegmen tympani on CT imaging.

The study was conducted on consecutive patients who visited the radiology departments of the aforementioned hospitals for CT scanning of their temporal bones for various reasons (hypoacusis, facial paralysis, vertigo, tinnitus, chronic otorrhea and otodynia). Patients with any type of anatomical alteration of the labyrinth and those with insufficient quality CT images to determine the studied parameters were excluded.

The absence of bone coverage of the tegmen tympani was assessed in coronal planes and was considered dehiscent when a lack of bone was observed between the middle ear/antra and the middle cranial fossa in one or more coronal CT slices, whether unifocal or multifocal. In the case of the superior semicircular canal, dehiscence was considered to exist when an opening was detected in the bone lining the canal in communication with the middle cranial fossa in reformatted CT slices in the plane of the canal or Pöschl's plane. In all the canals, the minimum thickness covering the roof was measured; dehiscence diagnosis required it to be present in least two consecutive images. In cases that were doubtful, patients underwent evoked potentials.

The degree of temporal pneumatisation was assessed by axial and coronal planes and divided into grades 0, I, II and III as follows:

***Grade 0***. Temporal sclerosis.

***Grade I***: Only the mastoid antrum and some adjacent cells are pneumatised, but there is no evidence of pneumatisation inside the bony labyrinth.

***Grade II***. Pneumatisation occupies part of the mastoid but not the mastoid apex. Cells are located in the lateral region of the superior semicircular canal but not in the carotid duct or in the tip.

***Grade III***. The pneumatisation occupies the entire mastoid with its apex and surpasses the semicircular canals (perilabyrinthine pneumatisation), the limits of the lateral sinus, and even surrounds the carotid canal. A certain degree of pneumatisation of the petrosal apex is also observed.

All the studies were conducted using Multislice Helical Computed Tomography equipment, obtaining images in the axial plane and with the patient's neck in hyperextension to avoid direct radiation of the crystalline lens. Subsequently, in all cases, coronal reconstructions and reconstructions in the plane of the superior semicircular canal of each ear were performed. The "raw data" were reconstructed using a bone algorithm.

The main variable of the study is "presence of both dehiscences" with a dichotomous response: Yes or No. The independent variables are "Age", "Sex" and "Degree of pneumatisation": grade 0, I, II, or III. For the statistical evaluation, only grades I, II and III were considered since no cases with sclerosed temporalis (Grade 0) were identified.

### Statistical method

First, the variables considered were described using absolute frequencies and percentages with a 95% confidence interval (CI).

In the bivariate analysis, if the variables were quantitative, they were tested for normality using the Kolmogorov‒Smirnov test. If they followed a normal distribution, the comparison was performed using Student's *t* test, and if they did not follow a normal distribution, the nonparametric Mann‒Whitney *U* test was used.

A Chi-square test was used for qualitative variables; Fisher’s exact test was applied if the cells were not well filled. The Haberman corrected standardised residuals were analysed to determine the relationship between the values of the variables.

In the multivariate analysis, as the distributions followed a normal distribution, a comparison was made using one-factor analysis of variance (ANOVA). Pairwise comparisons were conducted with Bonferroni correction because the data were homoscedastic (Levene test *p* < 0.05).

The data were processed with the SPSS 26.0 statistical package licenced by the University of Zaragoza, and the level of significance chosen for all the comparisons was 0.05.

Approval from each of these centres’ Ethics Committees was obtained, according to the guidelines of the 1964 Declaration of Helsinki.

## Results

We analysed 124 cases with a defect in the bony integrity of the tegmen tympani, investigating whether the superior semicircular canals were intact or dehiscent, to study the association between these structural defects. We also studied the type of pneumatisation of the temporal bone to determine whether any of the established degrees were significantly associated with having both tegmen and canal dehiscences.

The mean age of the 124 patients was 59.5 ± 13.7 years; 47 (37.9%) were men with a mean age of 61.3 ± 13.3 years, and 77 (62.1%) were women with a mean age of 58.4 ± 13.9 years. The difference of 2.8 years was not significant (*t*-Student = 1.14, *p* = 0.256) (Table 1).

**Table 1:** Age distribution by presence or absence of both dehiscences, sex and degree of pneumatisation

	Mean (SD*)	Median (IR**)	*p*
Presence of both dehiscences (number)			
Yes (35)	58.3 (13.3)	59.0 (21)	0.539^$^
No (89)	60.0 (13.9)	60.0 (21)	
** Total (124)**	**59.5 (13.7)**	**59.5 (21)**	
Sex (number)			
Male (47)	61.3 (13.3)	62 (25)	0.256^$^
Female (77)	58.4 (13.9)	59 (20)	
** Total (124)**	**59.5 (13.7)**	**59.5 (21)**	
Grade of pneumatisation (number)			
Grade I (27)	62.8 (16.8)	62 (18)	0.291^&^
Grade II (55)	59.5 (11.6)	59 (16)	
Grade III (42)	57.5 (14.0)	58 (24)	
** Total (124)**	**59.5 (13.7)**	**59.5 (21)**	

SD* Standard Deviation. **Interquartile Range. ^$^Statistic test: t-Student. ^&^Statistic test: ANOVA

When classified according to the degree of pneumatisation, grade I pneumatisation was observed in 27 (21.8%) cases, grade II in 55 (44.4%) and grade III in 42 (33.9%), with a mean age of 62.8 ± 16. 8 years in grade I cases, 59.5 ± 11.6 years in grade II cases and 57.5 ± 14.0 years in grade III cases. These differences did not reach significance (ANOVA F = 1.25, *p* = 0.291) (Table 1).

However, when relating the degree of pneumatisation to sex, among those with grade III pneumatisation, the proportion of men (52.4%) was significantly higher than that of women (47.6%) (*p* = 0.017).

Of the 124 cases studied, 35 (28.2%) presented both dehiscences, 3 (9.4%) (1 man and 2 women) showed dehiscences on both sides, and 29 patients (90.6%) presented dehiscences on only one side (13 left and 16 right). Of the 32 patients, 12 were asymptomatic, and 20 showed symptoms compatible with third window syndrome.

Regarding the presence of both dehiscences with age, the mean age of the cases with both dehiscences was 58.3 ± 13.3 years, and that of those without was 60 ± 13.9 years. The difference of 1.74 years was not significant (*t* = 0.616, *p* = 0.539) (Table 1).

Regarding the association between the main variable and sex, 13 (27.7%) of the 47 men and 22 (28.6%) of the 77 women presented both dehiscences. This difference was not significant (chi-square = 0.012; *p* = 0.913) (Table 2). Regarding the degree of pneumatisation, 4 (14,8%) of the 27 cases with grade I pneumatisation, 26 (47,3%) of the 55 cases with grade II pneumatisation and 58 (11,9%) of the 42 cases with grade III pneumatisation presented both dehiscences. This difference was significant (Chi-square = 17,769; *p* < 0,001).

**Table 2:** Relationship between the existence or not of both dehiscences with sex and degree of pneumatisation

	Presence of both dehiscence		*p*
	Yes number (%)	No number (%)	
Sex (number)			
Male (47)	13 (27.7)	34 (72.3)	0.913
Female (77)	22 (28.6)	55 (71.4)	
**Total (124)**	**35 (28.2)**	**89 (71.8)**	
Grade of pneumatisation (number)			
Grade I (27)	4 (14.8)	23 (85.2)	< 0.001
Grade II (55)	26 (47.3)	29 (52.7)	
Grade III (42)	5 (11.9)	37 (88.1)	
** Total (124)**	**35 (28.2)**	**89 (71.8)**	

Statistic test: Chi-square

Of the 35 cases with double dehiscence, 5 had extensive pneumatisation, and 4 of them were men; this association approached significance (Fisher's exact test *p* = 0.052, *p* = 0.052).

## Discussion

When evaluating our results, we obtained an incidence of dehiscent tegmens associated with dehiscence of the superior semicircular canal of 28.2%, a lower incidence than that described by Crovetto et al. (36.4%) [9], Whyte et al. (37.3%) [4], Formeister et al. (37.5%) [11], El Hadi et al. (56.5%) [12] and Nadaraja et al. (76%) [3] but higher than that of Cloutier et al. (13.04%) [2]. We think, as all of the above studies stated, that the association between both bone defects is the most frequent of all those that could coexist in the temporal bone. Thus, we assume that the discordance in the incidence data listed above could be due to the relatively low sensitivity of computed tomography for detecting tegmen dehiscence. An example of this is found in other publications, such as that of Formesiter et al., in which 19.9% of patients (27/136) were diagnosed with a tegmen dehiscence during surgery for superior semicircular canal dehiscence [11].

We agree with Fraile et al. that the high prevalence of this association is because both entities would have, in part, a common embryological origin since the primary centres of the superior semicircular canals collaborate in the ossification of the tympanic tegmen (tegmental prolongation) and the periosteum that separates the tegmen from the middle cranial fossa is a continuation of the one that covers the superior semicircular canal [13]. In 2016, Wackym et al. coined the term "otic capsule syndrome" to define the existence of associated dehiscences between any of the semicircular canals (superior, posterior or lateral) [14], and in 2020, Whyte et al. expanded this term to include multiple other dehiscences of structures derived from the otic capsule, such as the tegmen tympani, mastoid antrum, posterior semicircular canal, internal auditory canal or Fallopian aqueduct [15]. In 2023, Zarandy et al. studied the different types of dehiscences of the otic capsule and found a 6 and 2.7% prevalence of dehiscences of the superior and posterior semicircular canals, respectively; that of cochlear–facial dehiscence was 6.3%, that of the cochlea with the auditory canal was 0.7%, that of the cochlea with the carotid canal was 0.7%, that of dehiscence of the jugular bulb and aqueduct was 6.3% and that of the jugular bulb with the posterior semicircular canal was 0.2% [16]. When analysing this information, we found no mention of the link between tegmen dehiscence and superior semicircular canal dehiscence, despite this being the most prevalent presentation.

Although not significant, we observed more women with both dehiscences (22/35) than men (13/35). This female predominance has also been observed in, among others, the studies of Lookabaugh et al. [17], Stevens et al. [18], and Formeister et al. [11].

We disagree with Martin et al., who reported a higher frequency of bilateral bone defects (5/9) [19], since we observed only 3 patients with both bilateral dehiscences, while 29 had dehiscences on one side only.

Our literature review revealed no studies demonstrating a possible association between both dehiscences and the degree of pneumatisation of the temporal bone; thus, we examined whether there is a predominant pattern or degree of pneumatisation in these cases. To this end, we developed our own classification, including the states of the mastoid, petrous process and vestibule, with special emphasis on the connection of the pneumatised cells with the superior semicircular canal. Consistent with the studies by Crovetto et al. [9] and Tikka et al. [10], we detected no sclerosed temporal bone or grade 0 cases. In the latter two manuscripts and in that of Aladeyelu et al. [20], the relationship between pneumatisation of the temporal bone and dehiscences of the superior semicircular canal or the jugular gulf and carotid duct is mentioned but not the association of this pneumatisation with dehiscences of the tegmen and superior semicircular canal.

One difficulty in studying pneumatisation of the temporal bone is the existence of different classifications no consensus among researchers. In fact, some are limited to the mastoid [21, 22], and another is based on its relationship with the glenoid cavity [23]. Han et al. proposed three different classifications according to the relationship with the labyrinth, petrosal segment of the carotid canal and sigmoid sinus [24]. Pneumatisation has also been classified into poor, medium and extensive [9] or into hypopneumatisation, moderate pneumatisation, good pneumatisation and hyperpneumatisation [20]. These circumstances have led us to produce our own four-grade classification (0–3).

We found significance between moderate or grade II pneumatisation and the presence of both dehiscences (*p* < 0.001). These results are in agreement with the observations described by De Jong et al., who demonstrated a lower thickness and pneumatic density of the temporalis in patients with superior semicircular canal dehiscence than in controls with temporal bones without pathology [7]. Our findings are also consistent with Shim et al., who found a greater decrease in the volume of the mastoid cells in patients with superior semicircular canal dehiscences than in controls with otosclerosis or temporal bone fractures [8]. In contrast, in the analysis developed by Tikka et al., 83.3% of patients with superior canal dehiscence showed a more extensively pneumatised pattern than that described by us when the association was present, but only the mastoid was considered, not the location of the rest of the pneumatised cells. These authors also suggest that it would be reasonable to suppose that well-pneumatised temporals would have thinner bone over the superior semicircular canal and, therefore, would be more susceptible to a second event that would produce dehiscence [10]. These data differ from those provided by Cisneros et al., who observed that pneumatised superior semicircular canals are thicker, with a thickness greater than 2.5 mm, an average endosteum of 0.96 mm, periosteum varying between 0.5 and 1.2 mm, and pneumatic cells between both layers of 0.8 mm. These authors concluded that pneumatised canals have no clinical implications [25].

On evaluating the different degrees of pneumatisation in the cases demonstrating an association between canal and tegmen dehiscence by sex, we observed that in women, the majority of cases presented grade II pneumatisation, while men presented a near-significant relationship with grade III pneumatisation. These latter data could be explained by considering the results of previous studies, such as that of Castro Pérez et al., who observed a greater propensity for pneumatisation in men than in women, in whom the opposite phenomenon was observed [26], or Tan et al., who demonstrated that male sex was significantly associated with an increase in pneumatisation in the petrous apex and infralabyrinthine compartments (*p* = 0.001) [27].

Given the unspecificity of the clinical symptomatology of these patients and their great variability and polymorphism, we have not been able to relate the degree of pneumatisation with the existence of both dehiscences from a clinical point of view. This encourages us to continue working in this line of research in the future, increasing the sample size, and trusting that this manuscript will serve as a reference for other research groups working on dehiscences of this type, to look for a possible clinical implication, as our group will continue to do.

We can conclude that relationship between the coexistence of grade II pneumatisation and the presence of both dehiscences in the temporal bone is statistically significant.

## Data Availability

Data are available on request due to privacy or other restrictions.

## References

[1] Minor LB (2000). Superior canal dehiscence syndrome. Am J Otol.

[2] Cloutier JF, Bélair M, Saliba I (2008). Superior semicircular canal dehiscence: positive predictive value of high-resolution CT scanning. Eur Arch Otorhinolayngol.

[3] Nadaraja GS, Gurgel RK, Fischein NJ, Anglemyer A, Monfared A, Jackler RK, Blevins NH (2012). Radiographic evaluation of the tegmen in patients with superior semicircular canal dehiscence. Otol Neurotol.

[4] Whyte J, Tejedor MT, Fraile JJ, Cisneros A, Crovetto R, Monteagudo LV, Crovetto M (2016). Association between tegmen tympani status and superior semicircular canal pattern. Otol Neurotol.

[5] Handzel O, Brenner-Ullman A, Cavel O, Oron Y, Wasserzug O, Fliss DM, Ungar OJ (2018). Clinical implications of the association between temporal bone tegmen defects and superior semicircular canal dehiscence. Otol Neurotol.

[6] Barbara M, Margani V, Voltattorni A, Monini S, Covelli E (2022). Concomitant dehiscences of the temporal bone: a case-based study. Ear Nose Throat J.

[7] De Jong MA, Carpenter DJ, Kaylie DM, Piker EG, Frank-Ito DO (2017). Temporal bone anatomy characteristics in superior semicircular canal dehiscence. J Otol.

[8] Shim SB, Kang BC, Kim CH, Kim TS, Park HJ (2012). Superior canal dehiscence patients have smaller mastoid volume than age - and sex - matched otosclerosis and temporal bone fracture patients. Korean J Audiol.

[9] Crovetto M, Whyte J, Rodríguez OM, Lecumberri I, Martínez C, Eléxpuru J (2010). Anatomo-radiological study of the superior semicircular canal dehiscence: radiological considerations of superior and posterior semicircular canals. Eur J Radiol.

[10] Tikka T, Kontorinis G (2020). Temporal bone anatomy in superior semicircular canal dehiscence: a case control study on bone pneumatization and the level of middle cranial fossa. Otol Neurotol.

[11] Formeister EJ, Zhang LS, Dent J, Aygun N, Carey JP (2022). Predictive factors for concurrent tegmen dehiscence in superior canal dehiscence syndrome. Otol Neurotol.

[12] El Hadi T, Sobrentino T, Calmels MN, Fraysse B, Degune O, Marx M (2012). Spontaneous tegmen defect and semicircular canal dehiscence: same etiopathogenic entity?. Otol Neurotol.

[13] Fraile JJ, Cisneros AI, Obón J, Yus C, Crovetto R, Crovetto MA, Whyte J (2016). Ontogenetic explanation for tegmen tympani dehiscence and superior semicircular canal dehiscence association. Acta Otorrinolaringol Esp.

[14] Wackym PA, Balaban CD, Mackay HT, Wood SJ, Lundell CJ, Carter DM, Siker DA (2016). Longitudinal cognitive and neurobehavioral functional outcomes before and after repairing otic capsule dehiscence. Otol Neurotol.

[15] Whyte J, Cisneros AI, García-Barrios A, Fraile JJ, Whyte A, Crovetto R, Lahoz M (2019). Association between superior semicircular canal dehiscence and other dehiscences in temporal bone. Folia Morphol.

[16] Zarandy MM, Kouhi A, Emami H, Amirzargar B, Kazemi MA (2023). Prevalence of otic capsule dehiscence in temporal bone computed tomography scan. Eur Arch Otorhinolayngol.

[17] Lookabaugh S, Kelly HR, Carter MS, Niesten MEF, Mckenna ML, Curtin H, Lee DJ (2015). Radiologic classification of superior canal dehiscence: implications for surgical repair. Otol Neurotol.

[18] Stevens SM, Hock K, Samy RN, Pensak ML (2018). Are patients with spontaneous CSF otorrhea and superior canal dehiscence congenitally predisposed to their disorders?. Head Neck Surg.

[19] Martin C, Chahine P, Veyret C, Richard C, Prades JM, Pouget JF (2009). Prospective radiological study concerning a series of patients suffering from conductive or mixed hearing loss due to superior semicircular canal dehiscence. Eur Arch Otorhinolayngol.

[20] Aladeyelu OS, Olojede SO, Lawal SK, Mbatha WBE, Sibiya AL, Rennie CO (2023). Influence of pneumatization on morphology of temporal bone-related vasculatures and their morphometric relationship with ear regions: a computed tomography study. Sci Rep.

[21] Carter LC, Haller AD, Calamel AD, Pfaffenbach AC (1999). Zygomatic air cell defect (ZACD). Prevalence and characteristics in a dental clinic outpatient population. Dentomaxillofacial radiology..

[22] Virapongse C, Sarwar M, Bhimani S, Sasaki C, Shapiro R (1985). Computed tomography of temporal bone pneumatization: normal pattern and morphology. AJR.

[23] Faleh Al, Ibrahim ME (2005). A tomographic study of air cell pneumatization of the temporal components of the TMJ in patients with temporomandibular joint disorders. Egypt Dent J.

[24] Han SJ, Song MH, Kim J, Lee WS, Lee HK (2007). Classification of temporal bone pneumatization base on sigmoid sinus using computed tomography. Clin Radiol.

[25] Cisneros AI, Whyte J, Martínez C, Obón J, Whyte A, Crovetto R, Crovetto MA (2013). Radiological patterns of the bony roof of the superior semicircular canal. Surg Radiol Anat.

[26] Castro Pérez F, Jerez Cordobez M, Rodríguez Gómez I, Rodríguez González R, Márquez Márquez DR (2009). Neumatización petromastoidea: impacto de enfoque etiológico multifactorial. Rev Cienc Med Pinar Rio.

[27] Tan AD, Ng JH, Lim SA, Low DYM (2018). Classification of temporal bone pneumatization on high resolution computed tomography: prevalence patterns and implications. Otolaryngol Head Neck Surg.

